# Molecular Characterization of Ambiguous Mutations in HIV-1 Polymerase Gene: Implications for Monitoring HIV Infection Status and Drug Resistance

**DOI:** 10.1371/journal.pone.0077649

**Published:** 2013-10-17

**Authors:** Du-Ping Zheng, Margarida Rodrigues, Ebi Bile, Duc B. Nguyen, Karidia Diallo, Joshua R. DeVos, John N. Nkengasong, Chunfu Yang

**Affiliations:** 1 Division of Global HIV/AIDS, Center for Global Health, Centers for Disease Control and Prevention (CDC), Atlanta, Georgia, United States of America; 2 CDC-GAP Angola, Luanda, Angola; 3 CDC-GAP Botswana, Gaborone, Botswana; 4 Department of Health and Human Services/US CDC, Hanoi, Vietnam

## Abstract

Detection of recent HIV infections is a prerequisite for reliable estimations of transmitted HIV drug resistance (t-HIVDR) and incidence. However, accurately identifying recent HIV infection is challenging due partially to the limitations of current serological tests. Ambiguous nucleotides are newly emerged mutations in quasispecies, and accumulate by time of viral infection. We utilized ambiguous mutations to establish a measurement for detecting recent HIV infection and monitoring early HIVDR development. Ambiguous nucleotides were extracted from HIV-1 *pol-*gene sequences in the datasets of recent (HIVDR threshold surveys [HIVDR-TS] in 7 countries; n=416) and established infections (1 HIVDR monitoring survey at baseline; n=271). An ambiguous mutation index of 2.04×10^-3^ nts/site was detected in HIV-1 recent infections which is equivalent to the HIV-1 substitution rate (2×10^-3^ nts/site/year) reported before. However, significantly higher index (14.41×10^-3^ nts/site) was revealed with established infections. Using this substitution rate, 75.2% subjects in HIVDR-TS with the exception of the Vietnam dataset and 3.3% those in HIVDR-baseline were classified as recent infection within one year. We also calculated mutation scores at amino acid level at HIVDR sites based on ambiguous or fitted mutations. The overall mutation scores caused by ambiguous mutations increased (0.54×10^-2^3.48×10^-2^/DR-site) whereas those caused by fitted mutations remained stable (7.50-7.89×10^-2^/DR-site) in both recent and established infections, indicating that t-HIVDR exists in drug-naïve populations regardless of infection status in which new HIVDR continues to emerge. Our findings suggest that characterization of ambiguous mutations in HIV may serve as an additional tool to differentiate recent from established infections and to monitor HIVDR emergence.

## Introduction

With over 8.2 million HIV-infected patients on antiretroviral therapy (ART) in low- and middle-income countries at the end of 2011, emergence and transmission of HIV drug resistance (HIVDR) are ongoing public health challenges in the battle against HIV/AIDS [1-6]. Because of the incomplete suppression of viral replication by ART [2-4,7], even with the optimal adherence, HIVDR may still emerge in ART-patients; whereas under suboptimal ART situations, such as incomplete ART compliance and/or improper practice of regimen prescriptions, development of HIVDR could be enhanced [8]. Currently, mutations associated with HIVDR at 85 sites are identified, including 32 in reverse transcriptase (RT, 16 for nucleoside RT inhibitors [NRTIs] and 16 for non-nucleosides RTI [NNRTIs]), 36 in protease, seven in envelope, and 10 in integrase genes [9].

Estimates of transmitted HIVDR (t-HIVDR) and HIV incidence have been two important surrogates in measuring ART efficacy and prevention program effectiveness [10]. To assess the t-HIVDR in resource-limited countries, the World Health Organization (WHO) recommends conducting HIVDR threshold survey (HIVDR-TS) in recently HIV-infected populations enrolled by using WHO criteria [11,12]. Likewise, estimate of HIV incidence also requires recent infections that are determined by using serological assays with cross-sectional HIV-positive samples at population level [13-15]. Nevertheless, studies have indicated that these assays overestimated HIV incidences due to false classification of recent infections in certain populations and lack of validated standards in accurately distinguishing recent (within 1 year) from established infections [13,14,16].

HIV evolves rapidly attributed partially to the high error rate of its RT [17,18], and accumulates mutations at a certain rate over time [19]. This results in generating a large number of variants (quasispecies) in a host and increasing genetic diversity in a viral population [20]. By analyzing the genetic relationships among quasispecies, the dynamic evolutional pathway of a variant can be tracked on a time scale within and among hosts [21,22]. In the early stage of viral replication, HIV variants with new point mutations account for only a small proportion of the total wild-type populations, thus a particular point mutation at an allele is detected as a mixture along with the wild-type by conventional population-based (Sanger) sequencing, which is termed as ambiguous mutation/nucleotide. By applying the defined nucleotide substitution rate of HIV-1 [19,20], the quantified ambiguous mutations within a sequence could be used to estimate the duration of an HIV infection. Recent studies have demonstrated a constant increase of ambiguous nucleotides during the first 8 years of HIV infections at a rate of 0.2% per year in HIV subtype B *pol*-gene [23], and 0.45-0.5% of ambiguous nucleotides as a cutoff for distinguishing recent from established infections [23-25]. In this study, we characterized ambiguous nucleotides in non-B subtype sequences from eight population-based HIVDR-TS and HIVDR monitoring surveys and determined a predictive value for estimating HIV infection status using a molecular evolutional approach and compared ambiguous mutation evolving rate between sequences generated from non-B (A, C, and CRF01_AE) and B subtype viral strains, and evaluated the accuracy of WHO epidemiological criteria for the recent infection determination. We also used this approach to monitor HIVDR development at the early stage of HIV infections.

## Materials and Methods

### Data sources and types

Sequence data were from HIVDR-TS conducted in seven countries (Angola, Botswana, China, Kenya, Malawi, Tanzania, and Vietnam) (n=416) and from a baseline survey (n=271) in monitoring HIVDR development in patients commencing ART in Nigeria during 2005-2009 (Table 1). The detail demographic and clinical data of participants were previously described [26-32]. In brief, all patients in HIVDR-TS were enrolled as recent infections according to WHO criteria [11,12]. For those from Angola, Botswana, Kenya, Malawi and Tanzania, they were pregnant women who were <25 years old, attending antenatal clinics (ANC), diagnosed with HIV infections for the first time, and ART-naïve; and for those from China and Vietnam, they were individuals attending voluntary counseling and testing (VCT) sites and were partially intravenous drug users (IDU). The WHO criteria were designed to increase the likelihood of identifying recently infected individuals for the HIVDR-TS. The participants in baseline survey were patients eligible for ART according to the Nigeria national guidelines for HIV/AIDS care and treatment in adolescents and adults (CD4 ≤200 cells/µl, WHO stage III or IV or AIDS defined illness) [33], they were most likely established or chronic HIV-infected individuals. This dataset was used to compare to those of recent infections in the HIVDR-TS for ambiguous mutation calibration.

**Table 1:** Characteristics of sequence datasets and subtyping in partial HIV-1 *pol* gene.

Country^a^	Year	Data source	Infection route	Sequences	Subtype^b^						
					A	B	C	G	CRF01_AE	Others	Untypeable
Angola	2009	ANC (HIVDR-TS)	heterosexual	39			9	5		5	20
Botswana	2007	ANC (HIVDR-TS)	heterosexual	134			132*				2
China	2006	VCT (HIVDR-TS)	Multi-routes	45		8	3		20*	9	5
Kenya	2005-2006	ANC (HIVDR-TS)	heterosexual	33	19*		2			1	11
Malawi	2006	ANC (HIVDR-TS)	heterosexual	52			51*				1
Tanzania	2005-2006	ANC (HIVDR-TS)	heterosexual	45	14*		17			4	10
Vietnam	2006-2008	VCT (HIVDR-TS)	Multi-routes	68					68		
Nigeria	2008	ET (T1 baseline)	unknown	271	5		1	135			
Canada	2002-2008	Early infection	unknown	63		63*					

^a^ Country data used in this study were collected from HIV drug resistance threshold survey (HIVDR-TS), T1 baseline survey, and published data (24) respectively; Antenatal care (ANC), Voluntary counseling and testing (VCT), Eligible for treatment (ET); Numbers labeled with asterisk (*) were sequences selected to represent subtypes for statistical analysis, see Table 3; ^b^ HIV-1 subtypes were primarily determined using REGA HIV subtyping tool (http://www.bioafrica.net/rega-genotype/html/subtypinghiv.html).

We also included a dataset of subtype B sequences (n=63) from published resources [24] for the comparison of ambiguous mutation preference with our non-B subtype sequences. These sequences were generated from individuals who had been infected within 155 days confirmed by serological tests.

### DNA sequencing, genotyping and subtyping

 All partial *pol-*gene sequences were generated using a validated HIV-1 genotyping assay using a conventional population-based bi-directional sequencing procedure [34-36]. The lengths of sequences were 981 (HIVDR-TS data) and 1,002 (Baseline HIVDR monitoring survey data) nucleotides (nts) containing HIVDR mutation sites of protease and RT region [9].

Sequences were primarily subtyped using Stanford REGA HIV-1 Subtyping Tool version 2.0 (http://dbpartners.stanford.edu/RegaSubtyping/). The sequences used in this study included those published previously (JQ617150-JQ617250), and new submissions (JX083986-JX123826).

### Detection and analysis of ambiguous mutations

Ambiguous mutations, which consist of mixed nucleotides at a sequence position and named using the standard IUPAC ambiguous nucleotide codes, were determined and automatically called using customized software, Recall [37] when the sequencing signal intensity of the minor base was ≥20% of the major base signal at a nucleotide position on bi-directional sequences after subtracting background noise. Ambiguous mutations were extracted from each of sequences and tallied at country level. The mean of the ambiguous mutations was then calculated using the formula: M_AM_ = ∑ N_AM_/N (M_AM_: mean of ambiguous mutations per sequence; N_AM_: number of ambiguous mutations of a sequence; ∑N_AM_: sum of ambiguous mutations in a dataset, N: total number of sequences in the dataset). The index (I) of ambiguous mutations was calculated using the formulas: I_AM_ = N_AM_/Ls for an index at sequence level, or I_AM_ = M_AM_/Ls for an index at a dataset level (I_AM_: index of ambiguous mutations per site; Ls: length of a sequence by nucleotide), (note: Ls is 1/3 of full length when calculation was for 1st, 2nd, or 3rd codon position).

The composition of nucleotides or ambiguous nucleotides in a sequence dataset was obtained using BioEdit with the algorithm of base composition and mass export [38]. Ambiguous mutations were then stratified at 1st, 2nd, 3rd and all codon positions by dataset of threshold, baseline and Vietnam (VT), or by HIVDR and non-HIVDR sites based on the 2013 HIVDR List [9]. The index of ambiguous mutation was calculated using the same formulas as described previously.

 At the AA level, we scored 1 for a pure mutated AA and 0.5 for an ambiguous mutated AA because of its ambiguity, and calculated the total DR mutation score at each of the HIVDR sites [9] with the formula: DR mutation % = ([N_MAA_+N_AMAA_/2]/N_SEQ_)×100% (N_MAA_: number of mutated AA; N_AMAA_: number of ambiguous mutated AA).

### Recent infection determination

 Based on the estimated HIV nucleotide substitution rate of 2×10^-3^ nts per site per year [19,20], a sequence with ≤2 ambiguous mutations, or 2×10^-3^ ambiguous nucleotides per site, was considered to be derived from a subject who was infected within one year. In a dataset, the percentage of recent infections was assessed by calculating the proportion of sequences that had ≤2 ambiguous mutations.

### Statistical analysis

Statistical analyses were performed using IBM SPSS Statistics 20 (IBM), or otherwise were indicted. Data of non-normal distribution were determined by one-Sample Kolmogorov-Smirnov Test. Plot of dataset median, interquartile range (IQR), and range with and without outliers was made using online resource (http://www.physics.csbsju.edu/stats/). The overall significant difference of values in all datasets was determined by Kruskal-Wallis test, and the difference of pairwise comparison was determined by Mann-Whitney test when Kruskal-Wallis P value was <0.05. For multiple comparisons, the P values were corrected by the Bonferroni method.

### Ethics Statement

This is a data mining study based on the sequences generated from our previously published survey studies [26-32] in which all the surveys had been approved by the local Institutional Review Board (IRB) from Angola, Botswana, China, Kenya, Malawi, Tanzania, Vietnam, and Nigeria as well as the Associate Director for Science at the Center for Global Health of CDC, USA who determined that the anonymous specimen testing performed at CDC was a non-human subject research. 

## Results

### Range and index of ambiguous mutations for country-based datasets

**Figure 1:**
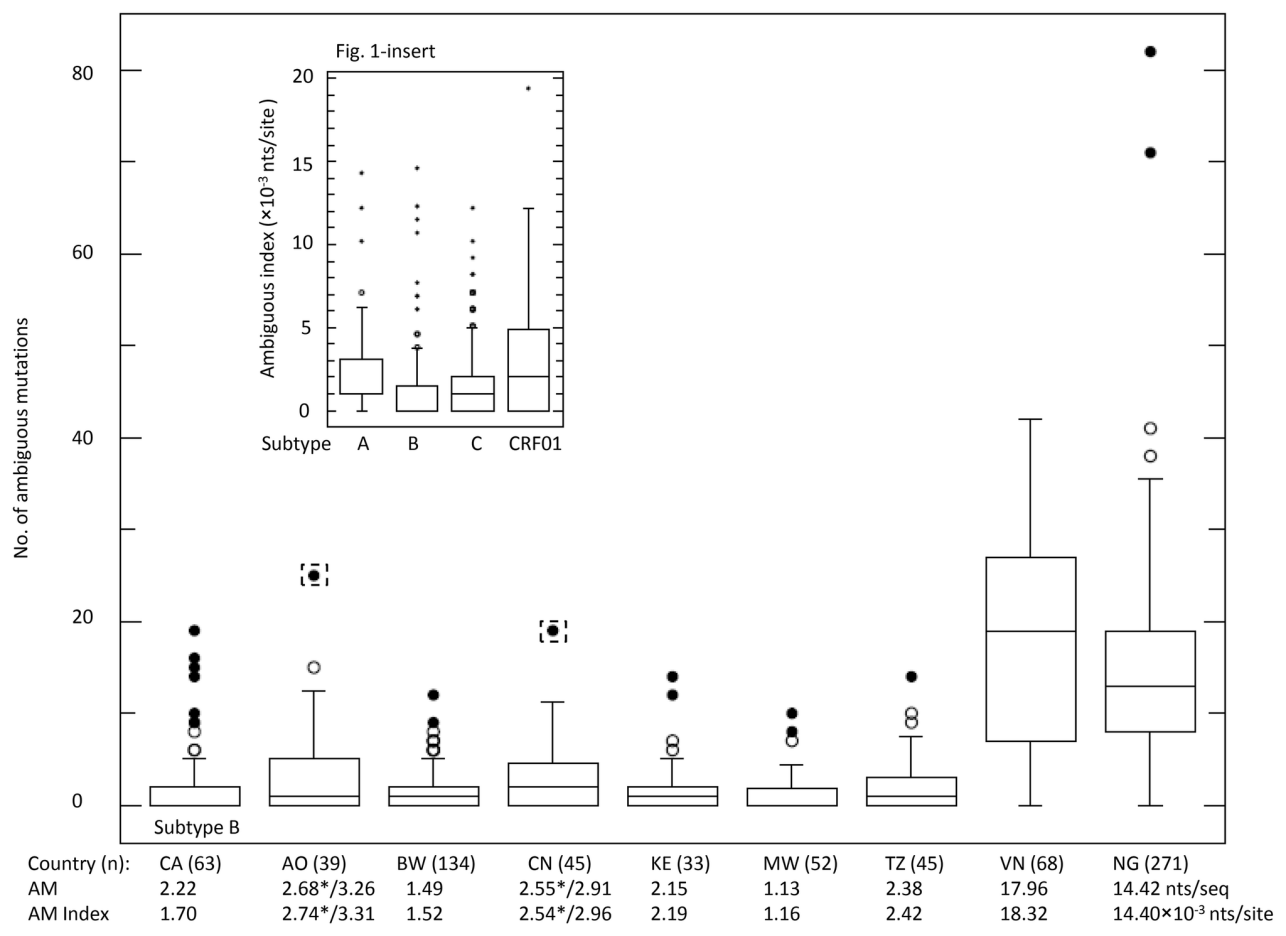
Descriptive statistics of ambiguous mutation in various sequence datasets. Plot of ambiguous mutations with descriptive statistics was performed using online statistical tool (http://www.physics.csbsju.edu/stats/). Individual country dataset was described for minimal and maximal ranges (short horizontal line at the bottom and top of the box), interquartile range (IQR, at 1^st^ to 3^rd^ quartile, box), median (line inside box), suspected outlier (open dot), and outlier (solid dot). Number in the bracket is the number of sequences from the country, Angola (AO), Botswana (BW), China (CN), Kenya (KE), Malawi (MW), Tanzania (TZ), Vietnam (VN), Nigeria (NG), and Canada (CA) [24]. Numbers with asterisk were calculated without the outlier in dash square box. Figure 1-insert shows the descriptive statistics of ambiguous mutation index in the dataset based on subtype (Table 3).

Ambiguous mutations were extracted from 8 datasets of a total of 687 sequences (Table 1) and were used for statistical descriptive and significant analyses (Figure 1). We observed that two HIVDR-TS datasets (Angola and China) each contained one sequence with much higher number of ambiguous mutations, 25 and 19, respectively than those in the remaining HIVDR-TS sequence datasets (Figure 1, indicated by dash box). For study purpose, we analyzed the two datasets with and without the outlier sequences.

For datasets from Angola, Botswana, China, Kenya, Malawi and Tanzania, the maximal range of ambiguous mutations without the two outliers was 0-15 nts with mean ranging from 1.13-2.68 nts per sequence. However, for those from Vietnam and Nigeria, the ranges were 0-38, and 0-82 nts with means of 17.96 and 14.42 nts per sequence, respectively. Likewise, the ranges of ambiguous mutation index were 1.16-2.74×10^-3^ nts per site among the datasets of Angola, Botswana, China, Kenya, Malawi and Tanzania; whereas the ranges of ambiguous mutation index for datasets from Vietnam and Nigeria were 18.32 and 14.40×10^-3^ nts per site, respectively (Figure 1).

When the two outliers were included in the analysis, the mean and index of ambiguous mutations were 3.26 nts per sequence and 3.31×10^-3^ nts per site for Angola, and 2.91 nts per sequence and 2.96×10^-3^ nts per site for China, respectively, which were slightly higher than the values generated without outlier sequences but they were still much lower than those from Vietnam and Nigeria (3.26 and 2.91 vs 17.96 and 14.42 for the mean of ambiguous mutations and 3.31 and 2.96 vs 18.32 and 14.40×10^-3^ nts per site for the index of ambiguous mutations, Figure 1).

Statistical analyses indicated that both mean and index of ambiguous mutation in the HIVDR-TS datasets (likely from recent infections) were significantly lower than those in the HIVDR monitoring baseline survey (established infection) (*p*<0.001) with an exception of those from Vietnam which is described in the later sections.

### Ambiguous mutation in recent and established HIV infected cohorts

**Figure 2:**
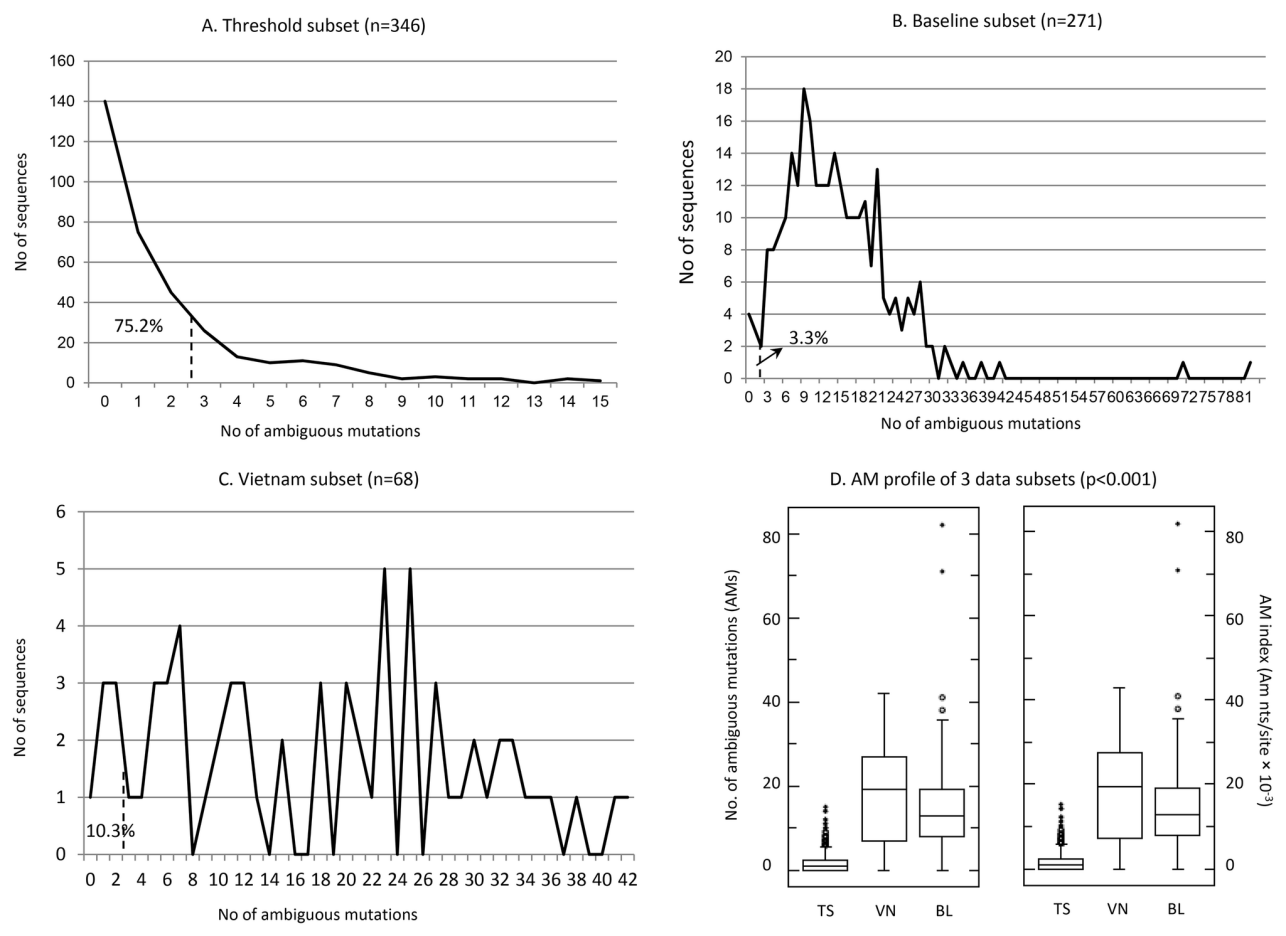
Distribution of ambiguous mutations and data statistical description of three data subsets. Sequence frequency distribution with number of ambiguous mutations (AMs) was plotted by subset: (A). Threshold (n=346), (B). Baseline (n=271), and (C). Vietnam (VN) (n=68); and the statistical description of the 3 data subsets was plot (D) by number and index of ambiguous mutations using the same method as described in Figure 1. The percentage in A-C indicated recent infections in a dataset classified by having ≤2 AMs per sequence (indicated by dash line).

To calibrate the difference of ambiguous mutations between recent and established HIV infections on a larger scale, we reorganized the data into three subsets: 1) threshold surveys for which samples were collected mainly from heterosexually transmitted individuals (N= 346), 2) baseline (N=271), and 3) Vietnam (N=68), and characterized the ambiguous mutations at each setting (Table 2). For the subset of threshold surveys, the mean of ambiguous mutations was 2.00 nts with a range of 0-15 nts per sequence, and the index was 2.04×10^-3^ (95% confidence intervals, [CI]: 1.13-2.71×10^-3^) ambiguous mutations per site. However, the subset from Vietnam, which was also from threshold surveys but might consist of subjects with different routes of transmission [30,39], and the one from Nigeria, which contained established and chronic infections, yielded means of 17.96 and 14.42 ambiguous mutations per sequence, and index of 18.30 (95% CI: 16.59-20.02) and 14.40 (95% CI: 13.50-15.29) ×10^-3^ ambiguous mutations per site (*p*=0.538), respectively, which were significantly higher than the values of the threshold data (*p*<0.001) (Table 2, Figure 2D). These combined data further confirmed that ambiguous mutation index was significantly lower in the individuals in the HIVDR-TS than those in the baseline and Vietnam surveys.

**Table 2:** Characteristics of ambiguous mutations (AMs) and rates between threshold and baseline sequence datasets.

	Threshold	Baseline	Vietnam-TS	*p*-value
Number of Sequences	346	271	117	
Sequence length (nts)	981	1002	867	
AMs and occurrence rate				
AM Range	0-15	0-82	0-38	
Mean (AMs/sequence)	2.00	14.42	12.78	
Rate (AMs/site, ×10^-3^) (95% C.I.)	2.04 (1.13-2.71)	14.40 (13.50-15.29)	14.74 (13.38-16.09)	<0.001 (TS vs BL, VN); 0.538 (VN vs BL)
AM occurrence rate (AMs/site, ×10^-3^)				
All codon positions				DR vs nDR: 0.889 (TS), 0.590 (BL), 0.441 (VN)
Non-DR sites (nDR)	2.02	14.97	14.35	
DR sites	2.07	12.09	12.26	
1^st^ codon position				
Non-DR sites	0.90	6.76	5.86	
DR sites	1.41	7.21	8.97	
2^nd^ codon position				
Non-DR sites	1.01	5.06	5.34	
DR sites	0.55	3.34	3.39	
3^rd^ codon position				
Non-DR sites	4.16	33.05	31.86	
DR sites	4.24	25.71	24.42	

^a^ The AMs of 2 outlier sequences were excluded for calculation.

We further characterized the sequence distribution by ambiguous mutations. Results exhibited completed different patterns between the 3 datasets (Figure 2A-C). In the threshold survey subset, the highest distribution of sequences peaked at 0 ambiguous mutation and 80% of the sequences had <3 ambiguous mutations; however, the peak of distribution curve shifted to the range of 3-28 ambiguous mutations in the baseline subset, and diversified with various cluster ranges of ambiguous mutation in the Vietnam subset (Figure 2A-C). These patterns of distribution curve indicated the uniformity of infection status among the subjects in the dataset: the narrower the distribution range is, the more uniformity the subjects are and a wide and diversified distribution pattern suggests a wide range of mixed infections, e.g. those shown in VN dataset. By using the estimated HIV nucleotide substitution of 2×10^-3^ nts per site per year [19,20], sequences of 75.2% in threshold, 3.3% in baseline, and 10.3% in Vietnam subsets could be classified as coming from people infected with HIV within one year. This result implies that around 75% of patients enrolled in the HIVDR-TS using WHO epidemiological criteria could be recently infected individuals.

### Ambiguous mutation at HIV drug resistant and non-resistant sites

To explore if there was any site preference for ambiguous mutation to occur, we measure the ambiguous mutation at DR or non-DR sites and all codon positions [9] . In general, no significant difference of ambiguous mutation was observed between DR and non-DR sites of all codon positions within each of the subsets (*p*=0.889 [HIVDR-TS], 0.590 [baseline], and 0.110 [VN-TS]) (Table 2), indicating a random mutation mechanism. However, at the 1st codon position the ambiguous mutation at DR sites was always higher than the non-DR sites across the datasets which was in reverse at the 2nd codon position. For example, in the threshold subset, the ambiguous mutation at the DR sites was 56.7% higher at the 1st codon position but 45.5% lower at the 2nd codon position than those at the non-DR sites. At the 3rd codon position, the ambiguous mutation between the DR and non-DR sites varied. They were similar in threshold (4.24 and 4.16), but were higher at the non-DR sites than the DR sites for the baseline and Vietnam subsets (33.05 vs 25.71; 42.50 vs 32.21, respectively), implying the evolutional pressure applied to DR and non-DR codon positions is different during the course of viral replication and/or infection.

### Amino acid fitness of ambiguous and mutated nucleotides at HIV DR sites

**Figure 3:**
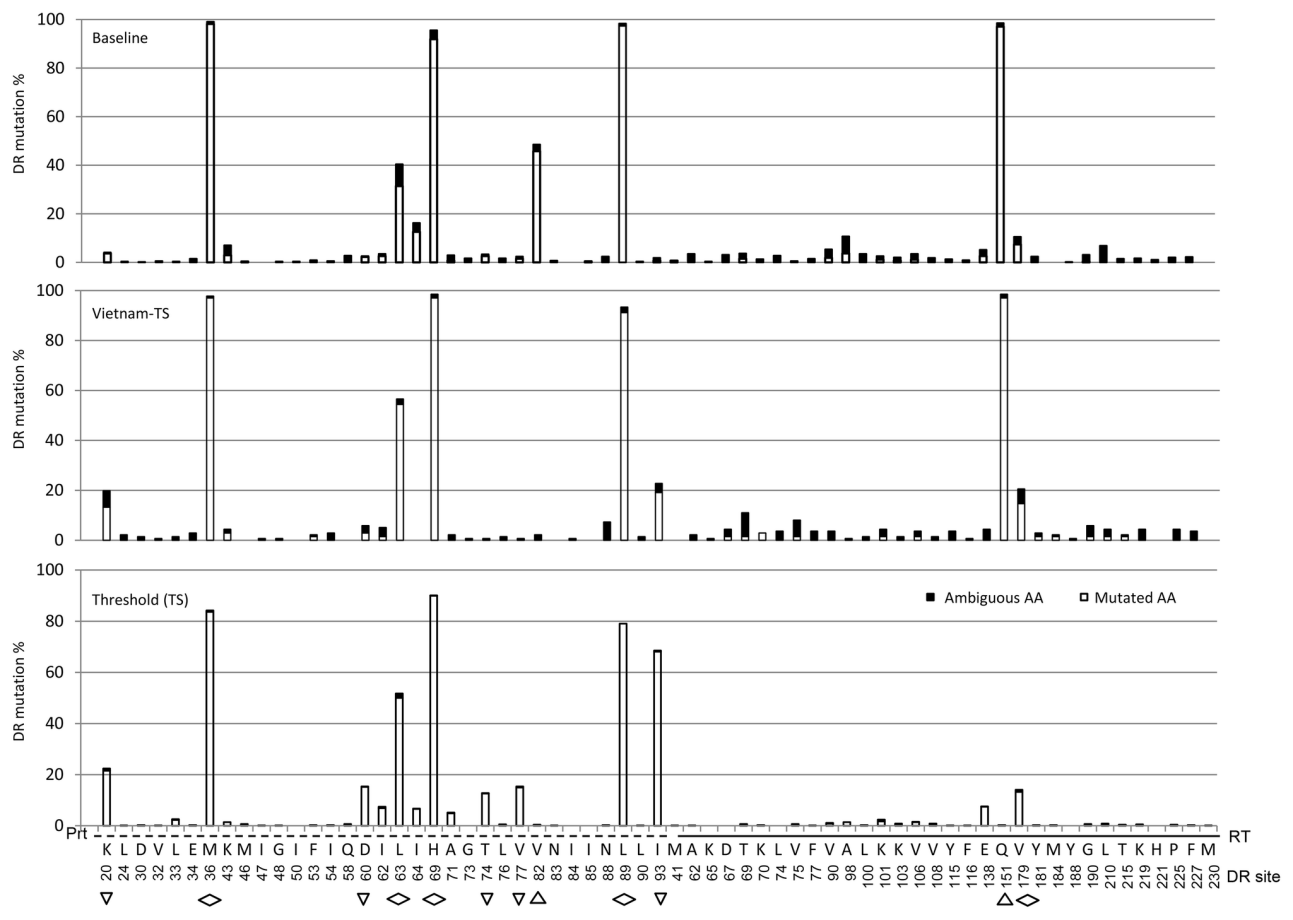
Proportional distribution of mutated and ambiguous mutated amino acids at HIVDR sites. The mutation score at each of the drug resistance sites [9] was proportionally calculated with the mutated and ambiguous mutated amino acids for all the sequences in the datasets. A mutated or ambiguous mutated amino acid was defined as an amino acid had mutated from a wild type to a pure non-synonymous mutation or an ambiguous mutation in the mixture allele. The scores were summed by 1 for a pure amino acid mutation and 0.5 for an ambiguous amino acid mutation, and then converted to percentages against the total number of wild-type amino acids at the site. The distribution of drug resistance mutation scores was plot by the dataset of Threshold (bottom panel), Vietnam (VN, central panel) and Baseline (top panel). The *x*-axis is the wild-type amino acids at drug resistance sites; the *y*-axis is the drug resistance mutation score (%). The sites with obvious score changes across the 3 datasets from bottom to top panel were labeled by up-triangle (increased), rhombus (remained), and down- triangle (decreased). Amino acids of protease gene (Prt) were top-dash lined, and of reverse transcriptase gene (RT) were top-solid lined.

To obtain the DR-score attributed to the ambiguous or non-ambiguous mutated nucleotides, we stratified the non-synonymous DR-associated mutations at amino acid (AA) level based on the 2013 HIVDR list [9] (Figure 3). Results showed that 12 of the 65 DR sites had patterns on the mutation score. It was constantly high at 3 DR sites (M36, 76.36-99.07%; H69, 90.10-98.53%; L89, 71.61-98.34%), moderate at 1 DR site (L63, 40.17-56.62%), and low at 1 DR site (V179, 10.52-20.59%) across all 3 subsets. The mutation score increased substantially at 2 DR sites (V82, 0.432.2148.52%; Q151, 0.2998.5398.52%), and decreased at 5 DR sites (K20, 20.3919.854.06%; D60, 15.375.882.58%; T74, 12.790.743.32%; V77, 15.370.742.40%; I93, 62.1522.791.85%) in the order of threshold, Vietnam and baseline subsets. Interestingly, of these 12 DR sites, 10 were located in the protease gene and only two (Q151 and V179) were in the RT gene. Some might associate with polymorphism or have combination effects on DR and viral replication depending on subtypes [40,41]. It was also evident that the DR score of ambiguous mutated AAs were gradually accumulating in the Vietnam and baseline sequences comparing to the threshold ones.

**Figure 4:**
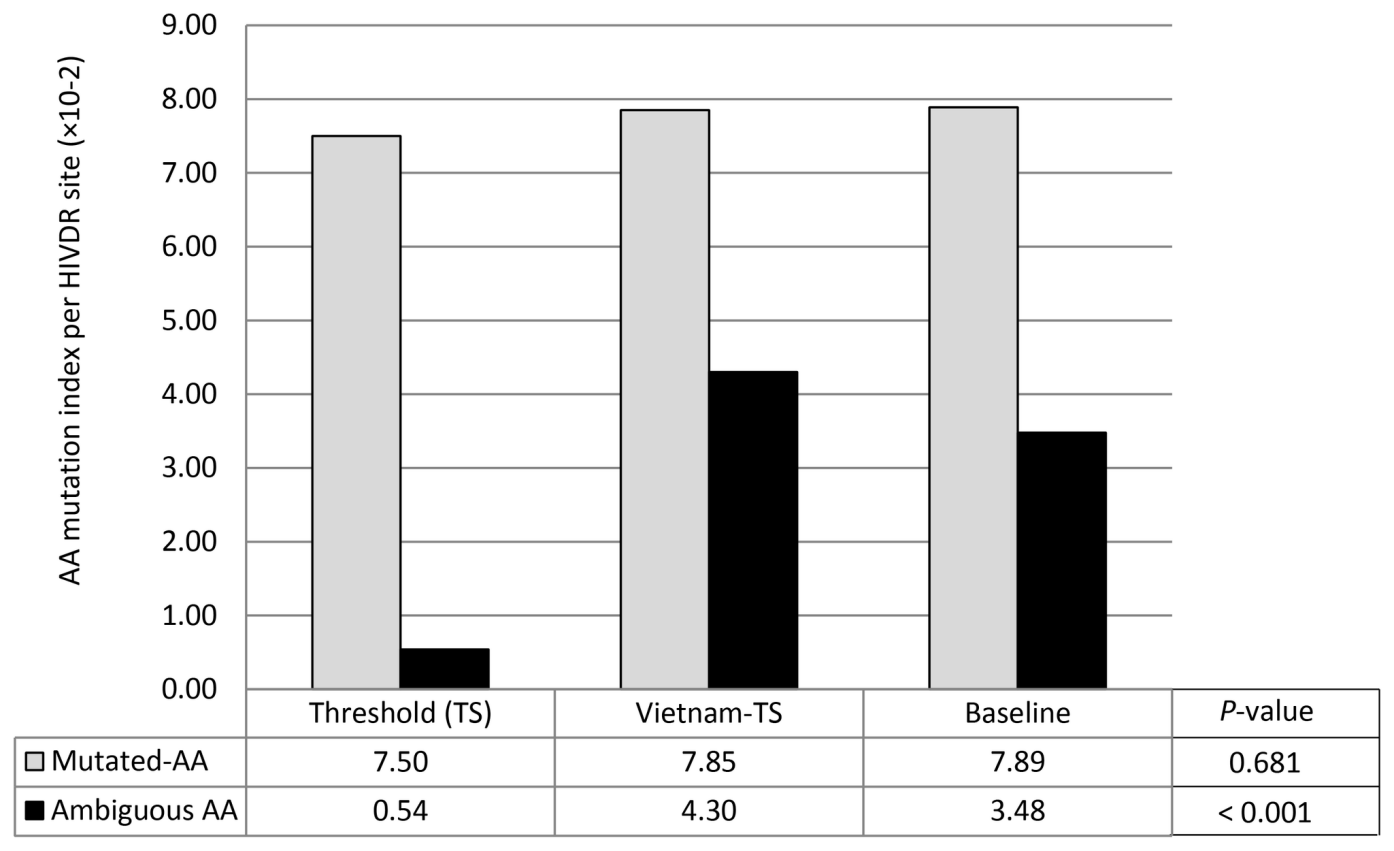
Index of mutated and ambiguous mutated amino acids at HIVDR sites by data subset. The total score of drug resistance mutations caused by pure mutated amino acids or by ambiguous mutated amino acids was calculated separately for each of the data subsets, and divided by the number of total drug resistance sites [9] to obtain the index of mutated or ambiguous mutated amino acids by subset. The definition and score calculation of pure mutated and ambiguous mutated amino acids were described in Figure 3.

By calculating the overall DR-associated mutations derived from pure mutated AAs or ambiguous mutated AAs (Figure 4), we found that the index of pure mutated AAs was constant across the 3 subsets (7.50-7.89×10^-2^ per DR site) (*p*=0.681); in contrast, the index of ambiguous mutated AAs increased significantly from 0.54×10^-2^ per DR site for threshold to 4.30-3.48×10^-2^ per DR site for Vietnam and baseline datasets (*p*<0.001), indicating that background of t-HIVDR existed in ART-naïve populations of these cohorts. Under such background, new HIVDR continued to develop in the studied populations.

### Subtyping and ambiguous mutation preference between subtypes

Subtype of HIV sequences in each dataset was primarily determined using the online REGA HIV subtyping tool (Table 1). A single dominant subtype was found in Botswana and Malawi (subtype C), Kenya (subtype A), Nigeria (subtype G), China and Vietnam (CRF01-AE); whereas multiple subtypes and recombinants were identified in Tanzania and Angola. Among those HIVDR-TS sequences, 46% were subtype C, 29% CRF01_AE, 7% subtype A and 10.5% untypeable.

To explore the difference of ambiguous mutation between subtypes, we collected a dataset of 63 subtype B sequences generated from specimens collected people infected with HIV-1 within 155 days [24], and compared them with all the non-B subtype or stratified non-subtype B sequences. These stratified sequences were selected from the dominant subtype(s) in the datasets, including subtype C from Botswana and Malawi, subtype A from Kenya and Tanzania, and CRF01_AE from China. Statistical analysis indicated that no significant difference of ambiguous mutation was found between subtype B and non-B subtypes (*p*=0.16) or subtype C (*p*=0.107); however, significant differences were noticed between subtypes B and A (*p*=0.001) or between subtype B and CRF01_AE (*p*=0.011, Table 3, Figure 1-insert).

**Table 3:** Comparison of ambiguous mutation (AM) in the recent infections of subtype B and other subtypes.

Subtype	Number of sequences	AM index (×10^-3^ nts/site)	95% CI	*P*-value
Non-B subtypes	346	2.03	1.62-2.21	0.16
A	33	2.55	1.59-3.52	0.001
B	63	1.71	1.01-2.40	
C	183	1.40	0.99-1.81	0.107
CRF01_AE	20	3.26	2.02-4.50	0.011

We couldn’t perform analysis of ambiguous mutation preference on the basis of infection route due to the incomplete infection route data from the China and Vietnam HIVDR-TS and the Nigeria baseline monitoring survey.

## Discussion

Detection of HIV recent infections is challenging and crucial for accurate HIV incidence and t-HIVDR estimations. We pursued an investigational molecular approach using ambiguous mutation for determining HIV infection status and monitoring early development of HIVDR. We characterized ambiguous mutations in recent (HIVDR-TS) and established infections (HIVDR baseline), and demonstrated the lower ambiguous mutation index was associated significantly with recent infections. We defined 2.04×10^-3^ ambiguous mutations/site as a measure for infections within one year, referred as recent infection in the current study. With the substitution rate defined, the proportion of subjects: 75.2% in the threshold, 3.3% in the baseline, and 10.3% in the Vietnam dataset, was classified being recent infections. These results provided data on the accuracy of defining HIV-1 recent infections using the WHO epidemiological criteria.

The dataset from Nigerian HIVDR monitoring baseline survey representing established infections exhibited a significantly higher mutation index (14.40×10^-3^ nts/site), which clearly differentiated them from the recent infections in the threshold subset, and served as a great calibrator for determining the ambiguous mutation index for recent infections. Based on our analyses, HIV infection status could range from 1 to 10 years for the majority of subjects in the Nigeria dataset, which reflects the reality because the subjects enrolled for ART included established and chronic infections according to the Nigeria HIV treatment guidelines [26,33]. The distribution curve of ambiguous mutations reflects the distribution of infection status of subjects in the dataset. For instance, a narrow sharp curve indicates a uniformity of subjects who had been infected around the same period of time whereas a wide curve indicates a wider range of infection status from recent to chronic infections. These could serve as a tool to evaluate uniformity of infection status in subjects from a dataset or cohort.

Although the Vietnam dataset was also from HIVDR-TS, only 10.3% of the subjects could be identified for being recently infected. The overall high ambiguous mutation index (18.32×10^-3^ nts/site) similar to the one found in the Nigeria baseline dataset was somewhat not surprising because in Vietnam the two highest HIV risk groups were IDUs and commercial sex workers [39,42] and the subjects in the Vietnam HIVDR-TS were enrolled at VCT [28,30]. In contrast, those from other HIVDR-TS surveys except for China were enrolled at ANC clinics. They were women attending their first pregnant visits and most likely infected through heterosexual transmission [27,29,31,32]. Because the mechanism of HIV evolution is transmission route dependent [2,21], IDUs have only about 40% of the chance being infected with a single virion and transmission of multi-viral strains through injection would amplify the founder effect in folds, leading to higher genetic diversities [20,43,44]. These might explain the Vietnam dataset and the two subjects with outlier sequences from China (6 IDUs were identified in the dataset, personal communication) and Angola. Thus, those with higher ambiguous mutations specifically in Vietnam dataset might not be established infections, but IDUs. 

Emergence and transmission of HIVDR is an on-going concern in the scale-up ART programs in resource-limited settings. To increase the elements of HIVDR surveillance and monitoring, we utilized the ambiguous mutation approach to monitor early emerged DR mutations and distinguish them from fitted DR mutations. Our data revealed an increase trend in DR mutations caused by ambiguous mutations (0.543.48×10^-2^) but a constant level of fitted DR mutations (7.50-7.89×10^-2^) from recent to established infections, indicating that t-HIVDR background exists in ART-naïve populations in which new HIVDR mutations continue to emerge over time. We also identified multiple DR sites that had mutational scores increased, remained stable or deceased over the time of infections. This dynamic distribution profile may be valuable for predicting early development of possible DRs in HIV-infected populations which provide data to decision maker for regimen considerations and/or selections.

Utilization of ambiguous mutations for predicting time of HIV infections may represent a relatively new sensitive approach. There are limited data available which were mainly focused on subtype B sequences, including two publications on HIV *pol-*gene [23-25] and one on *env-*gene [45]. The analyses focused on *pol-*gene found that >0.45-0.5% of ambiguous nucleotides provided strong evidence against a recent infection within 1 year and that ambiguous mutation rate constantly increased by 0.2% per year for the first 8 years of HIV infections. Our finding in which recent infections were defined as having an ambiguous mutation rate of 2.03×10^-3^ nts/site/year is in agreement with these studies and this is also corroborated with the substitution rate of 2×10^-3^ nts/site/year as reported by others [19,20]. 

Our observation on subtype preference in viral ambiguity showed no significant difference between subtype B and overall non-B subtypes (*p*=0.16), which is consistent with a recent study [25]. However, our subtype-stratified analyses appear to show somewhat different pictures since subtype A (*p*=0.001) and CRF01-AE (*p*=0.011) did show higher ambiguity when they were compared to subtype B. We believe that the discrepant results may be attributed to the smaller sample size that we have for these subtypes (Table 3). In this study, we classified 75.2% of subjects in the threshold surveys were infected within one year and this is in agreement with a recent study in which 73% of the pregnant women were identified as recent infections within one year from the datasets generated using the same WHO epidemiological criteria for identifying recent infections in resource-limited countries [25]. 

 However, there are limitations in our study. We characterized ambiguous mutations with datasets based on recent and established infections. However, due to the limited epidemiological data, we couldn’t conduct analysis and interpret the data based on transmission modes. We could only confirm the heterosexual transmission route for the women recruited at ANC clinics because they were all at their first pregnancy and diagnosed the first time with HIV infections. Therefore, the founder effect would likely be the viral replication mechanism, and the ambiguous mutation detected in the HIVDR-TS datasets by Sanger sequencing would reflect the genetic diversity in the viral population. However, for the dataset with suspected IDUs, Sanger sequencing might not be able to resolve the genetic diversity due to the multi-virion infection nature. With the progress on new generation sequencing technologies, e.g. deep or single genome sequencing, the multi-virion infection could be resolved at individual variant level to reflect the true genetic diversity [44-46]. We identified significant higher ambiguous mutations occurred in subtype A and CRF01_AE based on relatively small sample size. Studies on a larger number of these subtypes using epidemiologically defined cohorts of HIV infections would be useful to confirm our findings and further our understanding of ambiguous mutation preferences. Lastly, we detected an increase of ambiguous mutations at HIVDR sites. Due to the lack of clinical data, we couldn’t determine a threshold of early developed minor HIVDR mutations that would have clinical significance for treatment and regimen decision-making [3,47].

 In summary, we characterized ambiguous mutations in HIV-1 protease and reverse transcriptase gene regions with likely recent and established infections. We defined an ambiguous mutation index for detecting HIV recent infections and characterized the distribution of ambiguous mutations for monitoring the early development of HIVDR. Our data suggest that molecular characterization of ambiguous mutations in HIV-1 may serve as an additional tool along with serologic assays to differentiate recent from established infections, evaluate infection status, and monitor the early development of HIVDR.
